# Studies of Defect Structure in Epitaxial AlN/GaN Films Grown on (111) 3C-SiC

**DOI:** 10.3390/nano11051299

**Published:** 2021-05-14

**Authors:** Andreea Bianca Serban, Vladimir Lucian Ene, Doru Dinescu, Iulia Zai, Nikolay Djourelov, Bogdan Stefan Vasile, Victor Leca

**Affiliations:** 1 Doctoral School in Engineering and Applications of Lasers and Accelerators, University Politehnica of Bucharest, 060042 Bucharest, Romania; andreea.serban@eli-np.ro (A.B.S.); dd.doru@gmail.com (D.D.); 2 Extreme Light Infrastructure-Nuclear Physics (ELI-NP), “Horia Hulubei” National R&D Institute for Physics and Nuclear Engineering (IFIN-HH), 30 Reactorului Street, 077125 Măgurele, Romania; iulia.zai@eli-np.ro (I.Z.); nikolay.djourelov@eli-np.ro (N.D.); victor.leca@eli-np.ro (V.L.); 3 Department of Science and Engineering of Oxide Materials and Nanomaterials, Faculty of Applied Chemistry and Materials Science, University Politehnica of Bucharest, 060042 Bucharest, Romania; bogdan.vasile@upb.ro; 4 Faculty of Physics, University of Bucharest, 077125 Măgurele, Romania

**Keywords:** gallium nitride, epitaxial thin films, defect density, positron diffusion length

## Abstract

Several aspects such as the growth relation between the layers of the GaN/AlN/SiC heterostructure, the consistency of the interfaces, and elemental diffusion are achieved by High Resolution Transmission Electron Microscopy (HR-TEM). In addition, the dislocation densities together with the defect correlation lengths are investigated via High-Resolution X-ray Diffraction (HR-XRD) and the characteristic positron diffusion length is achieved by Doppler Broadening Spectroscopy (DBS). Moreover, a comparative analysis with our previous work (i.e., GaN/AlN/Si and GaN/AlN/Al_2_O_3_) has been carried out. Within the epitaxial GaN layer defined by the relationship $F\bar{4}3m$ (111) 3C-SiC || P63mc (0002) AlN || P63mc (0002) GaN, the total dislocation density has been assessed as being 1.47 × 10^10^ cm^−2^. Compared with previously investigated heterostructures (on Si and Al_2_O_3_ substrates), the obtained dislocation correlation lengths (*L*^e^ = 171 nm and *L*^s^ =288 nm) and the mean distance between two dislocations (*r*_d_ = 82 nm) are higher. This reveals an improved crystal quality of the GaN with SiC as a growth template. In addition, the DBS measurements upheld the aforementioned results with a higher effective positron diffusion length $L_{\mathrm{eff}}^{\mathrm{GaN}2}$ = 75 ± 20 nm for the GaN layer.

## 1. Introduction

Gallium nitride (GaN) is an important wide band-gap semiconductor used in the fabrication of nitride based heterostructure devices for photodetectors [1], electronics [2,3], and light emitting diodes [4,5] and is considered as a perspective material for positron moderation [6,7]. In this sense, understanding GaN’s defect structure becomes vital since any impurities, vacancy defects, or strains can influence its unique physical and optical characteristics [3,5,8].

Several growth techniques have recently been studied for the preparation of large-area GaN films but a major obstacle is still represented by the lack of a suitable material that could be used as a substrate for the nitrides while being compatible from both thermal and structural points of view [7,9]. The ideal solution would be the use of single-crystal GaN substrates but as of yet there is no viable method for producing such substrates with a low cost and on a large area, on account of the reduced nitrogen solubility and diffusion in liquid gallium [10]. Consequently, sapphire or silicon have been used as substrates for GaN epitaxy despite their respective limitations. For instance, although Si is used as a promising high-quality and low-cost substrate, it still presents a large lattice mismatch with GaN (17%) and a significant difference in thermal properties [11]. On the other hand, a low-temperature, thick AlN buffer layer must first be grown on sapphire substrates in order to attenuate the large lattice mismatch between GaN and Al_2_O_3_ [7,12].

Our previous studies revealed some differences in the influence of lattice mismatch between the substrates and the AlN/GaN layers [13,14]. However, the mechanisms through which the buffer layer relieves stress and how this consequently affects defect formation are difficult to control. Therefore, a proper substrate for GaN growth is still highly desired; preferably one that would be available in large quantities at a low cost, while also possessing matching dimensional lattice and thermal expansion coefficients. To this end, silicon carbide (3C-SiC, 6H-SiC) represents a suitable candidate for use as a substrate for GaN due to its reasonably low lattice mismatch and high electrical conductivity compared to the previously described substrates. Although far from achieving perfect heteroepitaxy, these qualities render SiC suitable enough to significantly reduce dislocation density [15,16].

The essential criteria for determining the suitability of a material as a substrate for GaN epitaxy is not only the lattice mismatch but also the material’s crystal structure, composition, roughness, and chemical and physical properties [17,18]. Thus, in order to get better quality GaN epitaxial layers, there is an increased interest in the substrates surface preparation [19] and deposition of the AlN or GaN low-temperature buffer layer [16,20]. Moreover, a recent study qualifies silicon carbide as being a proper substrate for GaN epitaxy, even without a buffer layer [21,22].

In the present paper, we report an analysis of the crystal quality of commercially available GaN epitaxial thin film grown on (111) 3C-SiC with an intermediate AlN buffer layer. The growth relationship between the substrate, buffer, and film, the consistency of the interfaces, dislocation densities, and characteristic positron diffusion length are all assessed via High-Resolution Transmission Electron Microscopy (HR-TEM), High-Resolution X-ray Diffraction (HR-XRD), and Doppler Broadening Spectroscopy (DBS) techniques.

## 2. Materials and Methods

The GaN/SiC sample used in this work with dimensions of 10 × 10 × 0.35 mm^3^ was produced at the NTT Advanced Technology Corporation (Kanagawa, Japan) for high-electron-mobility transistors. As presented in our previous work [13,14], the wafers’ exact growth conditions were not made available by the producer.

Three complementary techniques were applied for the characterization of the material in order to study the microstructure, the interfaces’ characteristics, and the defects distribution. The samples were investigated via HR-TEM, HR-XRD, and DBS. The HR-TEM measurements were carried out under 200 kV with a Cs-corrected Titan Themis 200 microscope (Thermo Fisher Scientific, Waltham, MA, USA) designed with a high-brightness electron source. Elemental line profiling was performed using a Scanning Transmission Electron Microscopy (STEM) detector and a Super-X Energy Dispersive Spectroscopy (EDS) detector for Selected Area Electron Diffraction (SAED). EDS data processing was performed using ImageJ software (National Institutes of Health and the Laboratory for Optical and Computational Instrumentation, Madison, WI, USA) [23], while SingleCrystal^®^ (Oxford, UK) and CrystalMaker^®^ (Oxford, UK) were used for simulating the SAED data and crystal structures [24].

The HR-XRD data were collected using a high-resolution Rigaku SmartLab X-ray diffractometer (Neu-Isenburg, Germany) featuring a 9 kW rotating anode (CuK_α_ = 1.5418 Å) and a high-resolution hybrid detector (HyPix-3000). The *ω* scans, taken at a fixed 2θ Bragg angle corresponding to selected (h k l) planes, were recorded in a double-axis configuration using Cross Beam Optics (CBO) and Ge (220) × 4 monochromator, resulting in an axial divergence of 0.003° in the vertical diffraction plane of the goniometer. In order to prevent the samples’ curvature from influencing the measurements, the incidence beam was limited by an incidence slit of 1 mm, while on the detector side 4 mm and 38.5 mm receiving slits were used (open detector configuration). The samples were aligned with the (111) plane of the SiC substrate prior to the measurement of the selected GaN planes in order to remove any asymmetry due to optics or sample misalignment.

DBS measurements were performed at room temperature using a beam of incident positrons with controllable energy at the slow positron beam line of the High Energy Physics Institute in Beijing, China. The intensity of slow positrons obtained by solid neon moderation was ~2 × 10^6^ s^−1^, forming a beam with a Ø 5.5 mm cross-section. A high purity Ge detector with an efficiency of 27% (model GEM20P4, ORTEC, Zoetermeer, The Netherlands) was located 20 cm away from the samples in a perpendicular orientation with respect to the axis of the positron beam. The approximate resolution of this detector was FWHM = 0.97 keV for the 511 keV line. Each spectrum was acquired over an 8 min cycle using fixed incident positron energy (*E*_+_) in the range of 0.5 to 25 keV. The resulting statistics of individual spectra is ~5 × 10^5^ counts for the 511 keV peak region.

## 3. Results and Discussion

**Figure 1:**
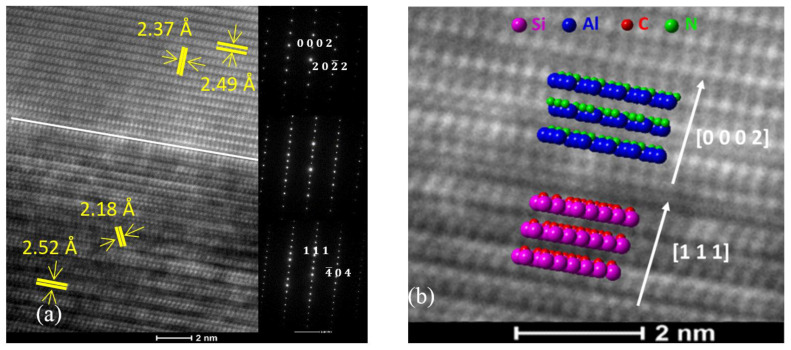
(**a**) HR-TEM micrographs and SAED patterns depicting the atom planes in SiC and AlN with respect to their interface; (**b**) HR-TEM micrographs overlaid with simulated crystal lattices near the interface between the SiC substrate and the AlN buffer layer.

The evaluation of the TEM cross-section images (Figure 1) reveals a buffer layer of aluminum nitride (AlN); therefore, the GaN/SiC sample can be regarded as a GaN/AlN/SiC heterostructure. Usually, such a layer aims at reducing the number of defects in the GaN film on account of the low lattice mismatch between AlN and GaN of 2.4% [25].

Figure 1a depicts HR-TEM micrographs and SAED patterns of the studied samples, revealing the arrangement of the SiC and AlN planes with respect to their interface. Below the interface, the planes of atoms parallel to the interface featured an interlayer distance of 2.52 Å, corresponding to the (111) lattice planes of $F\bar{4}3m$ cubic SiC (ICDD 00-029-1129). Additionally, the (200) lattice planes of cubic SiC were highlighted, showing an interlayer distance of 2.18 Å. Above the interface, the planes of atoms parallel to it featured an interlayer distance of 2.49 Å, which corresponds to the (0002) lattice planes of P63mc hexagonal AlN (ICDD 00-025-1133). Furthermore, interlayer distances of 2.37 Å were also measured, corresponding to the ($20\bar{2}2$) lattice planes of hexagonal AlN. The TEM analysis confirms epitaxial growth with the relationship $F\bar{4}3m$ (111) SiC ||P63mc (0002) AlN, as can be deduced using the overlays of simulated crystals in Figure 1b.

**Figure 2:**
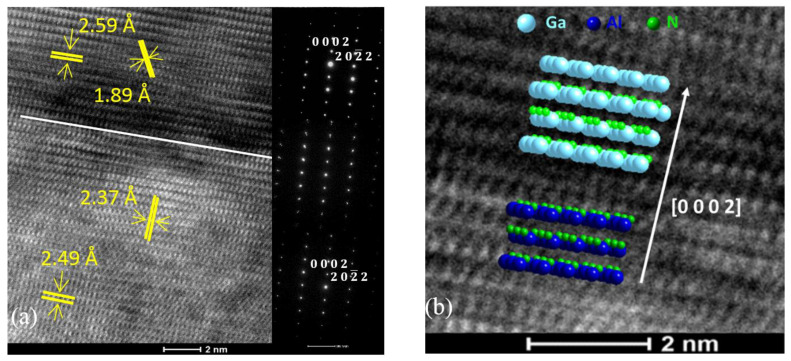
(**a**) HR-TEM micrographs and SAED patterns depicting the atom planes in AlN and GaN with respect to their interface; (**b**) HR-TEM micrographs overlaid with simulated crystal lattices near the interface between the AlN substrate and GaN buffer layer.

In Figure 2a, HR-TEM and SAED reveal the structure of AlN and GaN below and above their interface. Below the interface, the planes of atoms parallel to it show an interlayer distance corresponding to the (0002) lattice planes of P63mc hexagonal AlN. In addition, the ($20\bar{2}2$) lattice planes of hexagonal AlN are also depicted with an interlayer distance of 2.37 Å. Above the interface, the planes of atoms parallel to it featured an interlayer distance of 2.59 Å, which corresponds to the (0002) lattice planes of P63mc hexagonal GaN (ICDD 00-050-0792). Finally, interlayer distances of 1.89 Å were also measured, corresponding to the ($10\bar{1}2$) lattice planes of hexagonal GaN. It could thus be deduced that through a coherent interface, (0002) hexagonal GaN had grown over the (0002) hexagonal AlN with the relationship P63mc AlN (0002) || P63mc (0002) GaN, as simulated in Figure 2b.

**Figure 3:**
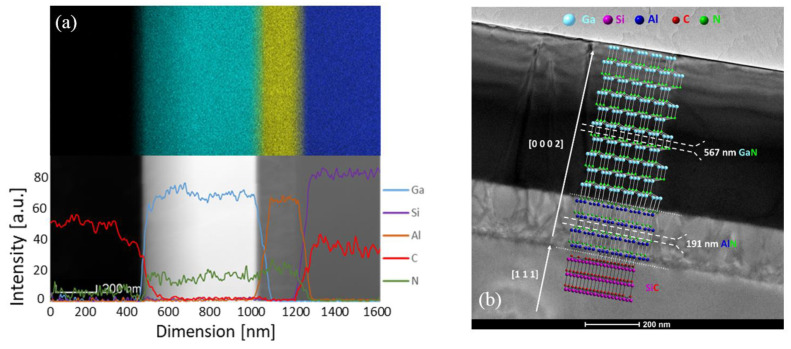
(**a**) STEM with EDS elemental mapping and line profile; (**b**) SiC/AlN/GaN sample overview with simulated crystal lattices.

The interface itself is barely noticeable and does not appear to show defects. The EDS data depicted in Figure 3a reveals negligible diffusion of any element outside of its layer boundary. The carbon present on the left of the elemental line profile belongs to the resin used for TEM sample preparation. On the 1 mm thick SiC substrate, layer thicknesses have been measured as 567 nm and 191 nm for the GaN film and the AlN interlayer, respectively. Figure 3b provides an overview of the sample, highlighting the thickness of the layers epitaxially grown by the relationship (111) SiC || (0002) AlN || (0002) GaN, with $F\bar{4}3m$ space group for the substrate and P63mc space group for the AlN and GaN layers, respectively.

**Figure 4:**
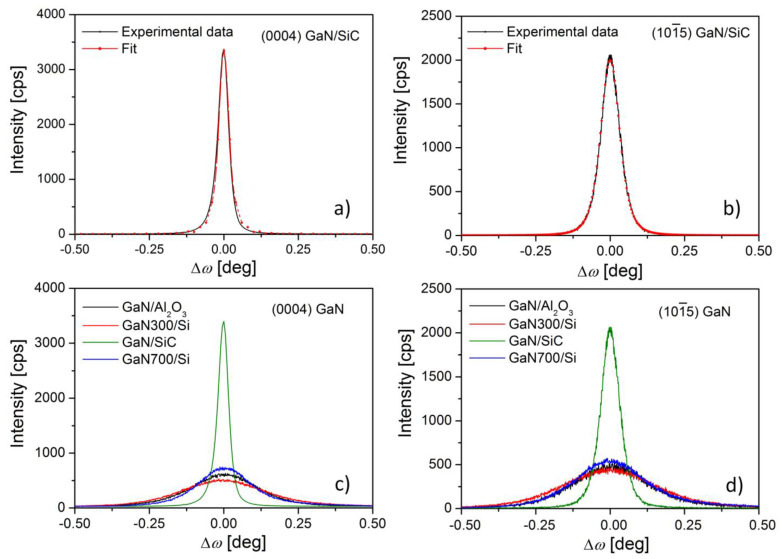
Omega scans (experimental and fits) around (0004) plane (**a**) and ($10\bar{1}5$) plane (**b**) of GaN films grown on (111) 3C-SiC substrate. For comparison we present in (**c**,**d**) the omega scans around (0004) and ($10\bar{1}5$) planes, respectively, for GaN films grown on (0001) Al_2_O_3_ and (111) Si substrates [13,14].

For the assessment of the threading dislocation density, $\rho_{d}$ and the correlation length, *L*, several *ω* scans (rocking curves) were performed for selected (h k l) planes (see Figure 4). In order to determine the edge dislocation density, $\rho_{d}^{e}$ and edge correlation length, $L^{e}$, the ($10\bar{1}5$) GaN plane was used, while for the screw values $\rho_{d}^{s}$ and $L^{s}$, the (0004) plane was measured.

The resulted rocking curve scans were processed applying the equation used by Kaganer et al. [26]:

(1) $$I\left(\omega \right)=\frac{I_{i}}{\pi }\int_{0}^{\infty }\exp(-Ax^{2}ln(\frac{B+x}{x}))\cos(\omega \cdot x)dx+I_{\mathrm{backgr}}$$

where *I*_i_ represents the integrated peak intensity, *I*_backgr_ represents the background intensity, *A* and *B* are parameters that describe the dislocation density and dislocation correlation range, respectively, and *x* is the arbitrary direction along which the correlations are measured. The dislocation density and correlation length are associated with the *A* and *B* parameters. Their values have been extracted from a nonlinear least square fit, minimizing the difference between the observed intensity and the calculated one. They are defined as

(2) $$A=f\cdot \rho_{d}\cdot b^{2};\;B=\frac{g\cdot L}{b}$$

where *b* is the Burgers vector with the chosen values of the edge *b*^e^ = 0.32 nm, while the screw *b*^s^ = 0.52 nm lengths were given by the lattice parameters *a* and *c* of nominally strain-free hexagonal GaN. The dimensionless parameters *f* and *g* depend on the skew geometry of the diffraction setup. They are defined as

(3) $$f^{e}=\frac{0.7\cos^{2}(\psi )\cos^{2}(\phi )}{4\pi \cos^{2}(\theta_{B})};f^{s}=\frac{0.5\sin^{2}(\psi )\cos^{2}(\phi )}{4\pi \cos^{2}(\theta_{B})};\;g^{e}=\frac{2\pi \cos(\theta_{B})}{\cos(\phi )\cos(\psi )};\;g^{s}=\frac{2\pi \cos(\theta_{B})}{\cos(\phi )\sin(\psi )}$$

where ψ represents the angle between the scattering vector and the sample surface, φ represents the angle between the sample surface and the incident vector, and $\theta_{B}$ represents the Bragg angle.

The obtained threading dislocation densities corresponding to the GaN layer grown on SiC are $\rho_{d}^{e}$ = 1.37 × 10^10^ cm^−2^ and $\rho_{d}^{s}$ = 1.07 × 10^9^ cm^−2^. Consequently, the total threading dislocation density calculated as the sum of those two component densities is $\rho_{d}^{t}$ = 1.47 × 10^10^ cm^−2^. With the latter value, the mean distance between two dislocations was determined as being 82 nm according to $r_{d}=1/{\left(\rho_{d}^{t}\right)}^{1/2}$ [27]. The values of the dislocation correlation lengths, i.e., $L^{e}$ = 171 nm and $L^{s}$ = 288 nm, extracted from the peak profiles, can be ascribed to the low lattice mismatch between the substrate and the film. The uncertainty of the presented values is within the least significant digit.

DBS is one of the most sensitive techniques used for the detection of open-volume defects in materials [28]. Whenever positrons are implanted into condensed matter, they annihilate with electrons in less than 10^−9^ s. While in a defect-free material the positrons are in a Bloch state, i.e., delocalized, in the presence of defects, both negatively charged and neutral, the positrons are trapped into them before their annihilation. Thus, the annihilation gamma-rays carry information of the electrons associated with the defects. [29]. This phenomenon will then cause narrowing in the DBS Spectrum. The shape of the broadened Doppler spectrum is characterized by the *S* and *W* parameters, which will ultimately enable the assessment of the aforementioned defects. The *S* and *W* parameters reflect the changes caused by the annihilation of positrons with electrons with low and high momentum, respectively. While higher *S* values imply greater positron entrapment within defects, the *W* parameter is influenced by annihilation of positrons with core electrons and is used to detect the presence of impurities [21,30].

The DBS data were analyzed using the VEPFIT software and characterized through the sharpness parameter, *S*, calculated as the ratio between the counts in the annihilation peak’s central area (|*E*_γ_−511 keV| < 0.78 keV) and the total peak counts (500–522 keV). Considering the available TEM information on the wafer’s structure, a positron implantation profile with different layer densities was used [31]:

(4) $$P_{\rho }\left(z_{\rho },E_{+}\right)=\rho \left(z_{\rho }\right)/\rho_{0}P\left(z,E_{+}\right)$$

with $z=\int_{0}^{z_{\rho }}\rho \left(\zeta \right)/\rho_{0}d\zeta$, where *z* represents the depth at which the positron is located and $\rho_{0}$ stands for the density of the substrate. For performing the data analysis, the densities of GaN, AlN, and SiC were chosen as 6.15 g cm^−3^, 3.26 g cm^−3^, and 3.21 g cm^−3^, respectively. When solving the positron transport problem, the implantation, diffusion, trapping, and annihilation of free positrons were considered. Positron surface trapping, epithermal positrons, thermal positrons diffusing back toward the surface, and positronium (Ps) emission were all integrated using the VEPFIT model:

(5) $$S\left(E_{+}\right)=S_{e}F_{e}\left(E_{+}\right)+S_{s}F_{s}\left(E_{+}\right)+\Sigma S_{i}F_{i}\left(E_{+}\right)$$

where *S*_e_, *S*_s_, and *S*_i_ represent the characteristic parameters corresponding to the surface annihilation of epithermal positrons (*S*_e_) and thermalized positrons (*S*_s_), along with the annihilation of thermalized positrons within the bulk of the virtually uniform *i*-th layer (*S*_i_). *F*_e_(*E*_+_) stands for the fraction of epithermal positrons annihilated at the surface, *F*_s_(*E*_+_) corresponds to the fraction of surface-annihilated thermalized positrons, and *F*_i_(*E*_+_) represents the fraction of epithermal positrons annihilated within the bulk of the *i*-th layer. The relative estimated triple state of Ps emitted from the surface is given by *F*_Ps_ (*E*_+_), which is the ratio between the valley area counts of the energy spectrum (400–500 keV) and the total peak counts. *F*_Ps_ (*E*_+_) was fitted concomitantly on the same VEPFIT model.

The effective positron diffusion length is one of the parameters derived from data analysis and is described as $L_{\mathrm{eff}}={\left[D^{+}/\left(k_{t}n_{t}+\lambda_{b}\right)\right]}^{1/2}$, where *D*^+^ represents the positron diffusion coefficient, *λ*_b_ corresponds to the bulk annihilation rate, *k*_t_ stands for the positron trapping rate, and *n*_t_ is the defect density. In order to not influence the uncertainty of the above parameters, the diffusion length of the substrate was set to 184 nm [32]. The thicknesses of the layers were also fixed to the values determined via a TEM analysis.

**Figure 5:**
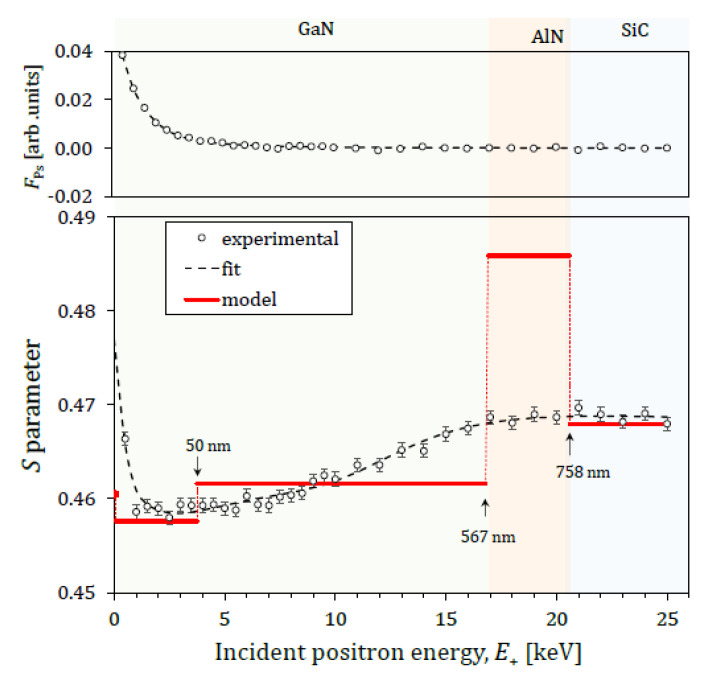
Plotted depth profiles *S*(*E*_+_) of GaN/SiC. The experimental errors are in the order of the experimental point size. The stairs represent the best parameters obtained by the fit of a 4-layer model to the experimental data by the VEPFIT software. The values represent the cumulative thickness of the layers. The upper part of figure is the experimental data and the best fit of the relative Ps fraction, *F*_Ps_(*E*_+_).

Figure 5 shows the depth profiles *S*(*E*_+_) and *F*_Ps_(*E*_+_) obtained for the GaN/AlN/SiC heterostructure along with the layer boundary depths calculated using the mean penetration depth *z*_m_ = (36/*ρ*)$E_{+}^{1.62}$. The best fit results are shown in Table 1. Our attempts to fit the experimental data using a three-layer model resulted in physically incorrect data, so a four-layer model was proposed instead, thereby splitting the GaN film into two sublayers (i.e., GaN1 and GaN2). A detailed explanation why the three-layer model results are incorrect is given in references [13,14].

According to Figure 5, a strong decrease in *S* was observed for *E*_+_ ≲ 1 keV due to the reduced ability of thermalized positrons to diffuse back toward the surface. This phenomenon is induced by the local electric field resulting from the band bending near the surface. Consequently, less Ps emission takes place at the surface and thus the positron diffusion length is reduced. At *E*_+_ ≳ 1 keV, *S* increases slowly with *E_+_* in the GaN film area, then seemingly reaches a plateau in the AlN layer (for *E*_+_ ≳ 17 keV), before finally achieving full saturation at *E_+_* ≳ 21 keV within the SiC substrate layer.

Since Ps is not formed within the bulk GaN layers, the inequality *S*_GaN1_ = 0.4576 ± 0.0004 < S_GaN2_ = 0.4615 ± 0.0004 indicates either a smaller (in size) or lesser number of defects as well as a longer *L*_eff_ within the GaN1 sublayer [33]. Given the resulting disagreement in $L_{\mathrm{eff}}^{\mathrm{GaN}1}$ = 13 ± 0.4 nm being smaller than $L_{\mathrm{eff}}^{\mathrm{GaN}2}$ = 75 ± 20 nm, the underlying reason could be related to the presence of a local electric field directed toward the surface.

The results which summarize the characteristics of the GaN layers listed in this study along with previously reported results [13,14] are shown in Table 2. Additionally, Figure 4c,d show the omega scans for (0004) and ($10\bar{1}5$) planes for GaN films grown on (111) 3C-SiC, (0001) Al_2_O_3_, and (111) Si substrates [13,14]. Comparing the results, one can observe that GaN exhibits an improved crystal quality in the GaN/SiC sample, with the lowest structural defect density of $\rho_{d}^{t}$ = 1.47 × 10^10^ cm^−2^ and the highest value of $L_{\mathrm{eff}}^{\mathrm{GaN}2}$ = 78 ± 20 nm. All of the investigated samples (i.e., GaN/Al_2_O_3_ [13], GaN300/Si, GaN700/Si [14], and GaN/SiC) demonstrated shorter effective positron diffusion lengths than that of defect-free GaN (i.e., $L_{\mathrm{eff}}^{\mathrm{DF}}$ = 135 nm), with the closest value being obtained for GaN/SiC. It is important to note that the GaN/Al_2_O_3_ mean distance between two dislocations was calculated within this paper and the positron data was revised by considering the relative estimated triple state of Ps emitted from the surface.

The smallest mean distance between two dislocations, $r_{d}=15$ nm, which is a consequence of the high value of the GaN300/Si wafer dislocation density ($\rho_{d}^{t}=4.37\times 10^{11}$ cm^−2^), implies a lower quality of the GaN layer as compared to the GaN/Al_2_O_3_, GaN700/Si, and GaN/SiC wafers. The conclusion of the lower crystal quality is also sustained by the linear dislocations and Al diffusion [14] near the AlN/GaN interface, as well as by the highest FWHM in omega scans for this GaN300/Si layer, as shown in Figure 4c,d. Even though GaN700/Si displays more point defects and linear dislocations at the interface, the decreased lengths of elemental diffusion relative to the GaN layer thickness induces a better quality of the top film, lowering the amount of threading dislocation densities [14].

## 4. Conclusions

Three methods were used for evaluating the relationship between the substrate, buffer, and film heterostructure and the consistency of its interfaces, layer thicknesses, dislocation densities, and positron-characteristic diffusion lengths in the GaN/AlN/SiC heterostructure. Edge and screw dislocations were assessed within the epitaxially grown layers defined by the relationship P63mc (0002) GaN || P63mc (0002) AlN || $F\bar{4}3m$ (111) 3C-SiC. The total dislocation density has been assessed as being 1.47 × 10^10^ cm^−2^. Compared with previously investigated heterostructures (on Si and Al_2_O_3_ substrates), the obtained dislocation correlation lengths (*L*^e^ = 171 nm and *L*^s^ = 288 nm) and the mean distance between two dislocations (*r*_d_ = 82 nm) are higher. This reveals an improved crystal quality of the GaN with SiC as a growth template. In addition, the DBS measurements upheld the aforementioned results, with higher effective positron diffusion length $L_{\mathrm{eff}}^{\mathrm{GaN}2}$ = 75 ± 20 nm for the GaN layer.

## Figures and Tables

**Table 1:** Best fit parameters obtained via VEPFIT software using the *S*(*E*_+_) and *F*_Ps_(*E*_+_) depth profiles. Values without any error margins represent fixed parameters.

Sample	GaN/SiC	*S*	*χ*^2^ = 1.18
Layer/Sublayer	*L*_eff_ [nm]		*d* [nm]
GaN1 GaN2	13.0 ± 0.4 75 ± 20	0.4576 ± 0.0004 0.4615 ± 0.0004	50 517
AlN	25 ± 18	0.4813 ± 0.0027	191
SiC	184	0.4680 ± 0.0008	-

**Table 2:** Total defect densities, defect correlation lengths, and effective positron diffusion lengths in GaN, where *d* is the layer thickness, $\rho_{d}^{t}$ is the total threading dislocation density, *r*_d_ is the mean distance between two dislocations, *L* is the defect correlation length (edge and screw), and *L*_eff_ is the effective positron diffusion length.

Sample	*d*_GaN_ [nm]	$\rho_{d}^{t}\;[\mathrm{cm}{-}^{2}]$	$r_{d}\;[\mathrm{nm}]$	*L* [nm]		*L*_eff_ [nm]	
				$L^{e}$	$L^{s}$	GaN1	GaN2
GaN/Al_2_O_3_ [18]	189	7.49 × 10^10^	36	155	229	12.4 ± 0.4	56 ± 4
GaN300/Si [17]	350	4.37 × 10^11^	15	27	107	14.3 ± 0.5	22 ± 6
GaN700/Si [17]	690	2.35 × 10^11^	21	41	220	13.1 ± 0.4	43 ± 6
GaN/SiC	567	1.47 × 10^10^	82	171	288	13.0 ± 0.4	78 ± 20

## Data Availability

Not applicable.

## References

[1] Wu H., Sun Y., Lin D., Zhang R., Zhang C., Pan W. (2009). GaN nanofibers based on electrospinning: Facile synthesis, controlled assembly, precise doping, and application as high performance UV photodetector. Adv. Mater..

[2] Hu H., Tang B., Wan H., Sun H., Zhou S., Dai J., Chen C., Liu S., Guo L.J. (2020). Boosted ultraviolet electroluminescence of InGaN/AlGaN quantum structures grown on high-index contrast patterned sapphire with silica array. Nano Energy.

[3] Hautakangas S., Makkonen I., Ranki V., Puska M.J., Saarinen K., Look D.C. (2006). Direct evidence of impurity decoration of Ga vacancies in GaN from positron annihilation spectroscopy. Phys. Rev. B.

[4] Zhou S., Liu X., Yan H., Chen Z., Liu Y., Liu S. (2019). Highly efficient GaN-based high-power flip-chip light-emitting diodes. Opt. Express.

[5] Liliental-Weber Z., Tomaszewicz T., Zakharov D., Jasinski J., O’Keefe M.A. (2004). Atomic structure of defects in GaN:Mg grown with Ga polarity. Phys. Rev. Lett..

[6] Uedono A., Sakurai H., Narita T., Sierakowski K., Bockowski M., Suda J., Ishibashi S., Chichibu S.F., Kachi T., Morkoç H., Fujioka H., Schwarz U.T. (2021). Behaviors of vacancy-type defects in Mg-implanted GaN during ultra-high-pressure annealing studied by using a monoenergetic positron beam. Proceedings of the Gallium Nitride Materials and Devices XVI.

[7] Lin M.E., Sverdlov B., Zhou G.L., Morkoç H. (1993). A comparative study of GaN epilayers grown on sapphire and SiC substrates by plasma-assisted molecular-beam epitaxy. Appl. Phys. Lett..

[8] Li B., Peng D., Li J., Kang L., Zhang T., Zhang Z., Jin S., Cao X., Liu J., Wu L. (2021). Magnetic and structural properties of Fe-implanted GaN at room temperature. Vacuum.

[9] Tang B., Hu H., Wan H., Zhao J., Gong L., Lei Y., Zhao Q., Zhou S. (2020). Growth of high-quality AlN films on sapphire substrate by introducing voids through growth-mode modification. Appl. Surf. Sci..

[10] Richter E., Hennig C., Weyers M., Habel F., Tsay J.D., Liu W.Y., Brückner P., Scholz F., Makarov Y., Segal A. (2005). Reactor and growth process optimization for growth of thick GaN layers on sapphire substrates by HVPE. J. Cryst. Growth.

[11] Takeuchi T., Amano H., Hiramatsu K., Sawaki N., Akasaki I. (1991). Growth of single crystalline GaN film on Si substrate using 3C-SiC as an intermediate layer. J. Cryst. Growth.

[12] Romanitan C., Mihalache I., Tutunaru O., Pachiu C. (2021). Effect of the lattice mismatch on threading dislocations in heteroepitaxial GaN layers revealed by X-ray diffraction. J. Alloys Compd..

[13] Ene V.L., Dinescu D., Zai I., Djourelov N., Vasile B.S., Serban A.B., Leca V., Andronescu E. (2019). Study of Edge and Screw Dislocation Density in GaN/Al2O3 Heterostructure. Materials.

[14] Ene V.-L., Dinescu D., Djourelov N., Zai I., Vasile B.S., Serban A.B., Leca V., Andronescu E. (2020). Defect Structure Determination of GaN Films in GaN/AlN/Si Heterostructures by HR-TEM, XRD, and Slow Positrons Experiments. Nanomaterials.

[15] Mu F., Cheng Z., Shi J., Shin S., Xu B., Shiomi J., Graham S., Suga T. (2019). High Thermal Boundary Conductance across Bonded Heterogeneous GaN–SiC Interfaces. ACS Appl. Mater. Interfaces.

[16] Saha S., Kumar D., Sharma C.K., Singh V.K., Channagiri S., Sridhara Rao D.V. (2019). Microstructural Characterization of GaN Grown on SiC. Microsc. Microanal..

[17] Kukushkin S.A., Osipov A.V., Bessolov V.N., Medvedev B.K., Nevolin V.K., Tcarik K.A. (2008). Substrates for epitaxy of Gallium Nitride:new materials and techniques. Rev. Adv. Mater. Sci..

[18] Liu L., Edgar J.H. (2002). Substrates for gallium nitride epitaxy. Mater. Sci. Eng. R Rep..

[19] Davis R.F., Bishop S.M., Mita S., Collazo R., Reitmeier Z.J., Sitar Z. (2007). Epitaxial Growth of Gallium Nitride.

[20] Fritze S., Drechsel P., Stauss P., Rode P., Markurt T., Schulz T., Albrecht M., Blsing J., Dadgar A., Krost A. (2012). Role of low-temperature AlGaN interlayers in thick GaN on silicon by metalorganic vapor phase epitaxy. J. Appl. Phys..

[21] Peng C.X., Weng H.M., Zhu C.F., Ye B.J., Zhou X.Y., Han R.D., Fong W.K., Surya C. (2007). Influence of GaN polarity and intermediate-temperature buffer layers on strain relaxation and defects. Phys. B Condens. Matter.

[22] Jarndal A., Arivazhagan L., Nirmal D. (2020). On the performance of GaN-on-Silicon, Silicon-Carbide, and Diamond substrates. Int. J. RF Microw. Comput. Eng..

[23] Schneider C.A., Rasband W.S., Eliceiri K.W. (2012). NIH Image to ImageJ: 25 years of image analysis. Nat. Methods.

[24] Palmer D.C. (2014). CrystalMaker.

[25] Lahrèche H., Vennéguès P., Tottereau O., Laügt M., Lorenzini P., Leroux M., Beaumont B., Gibart P. (2000). Optimisation of AlN and GaN growth by metalorganic vapour-phase epitaxy (MOVPE) on Si(111). J. Cryst. Growth.

[26] Kaganer V.M., Brandt O., Trampert A., Ploog K.H. (2005). X-ray diffraction peak profiles from threading dislocations in GaN epitaxial films. Phys. Rev. B Condens. Matter Mater. Phys..

[27] Romanitan C., Gavrila R., Danila M. (2017). Comparative study of threading dislocations in GaN epitaxial layers by nondestructive methods. Mater. Sci. Semicond. Process..

[28] GREEN J., LEE J., Hautojärvi P. (1964). Positrons in Solids. Positronium Chemistry.

[29] Uedono A., Ishibashi S., Oshima N., Suzuki R. (2013). Positron annihilation spectroscopy on nitride-based semiconductors. Jpn. J. Appl. Phys..

[30] Uedono A., Ishibashi S., Ohdaira T., Suzuki R. (2009). Point defects in group-III nitride semiconductors studied by positron annihilation. J. Cryst. Growth.

[31] Van Veen A., Schut H., de Vries J., Hakvoort R.A., Ijpma M.R. (1991). Analysis of positron profiling data by means of “VEPFIT”. AIP Conf. Proc..

[32] Uedono A., Itoh H., Ohshima T., Aoki Y., Yoshikawa M., Nashiyama I., Okumura H., Yoshida S., Moriya T., Kawano T. (1996). Defects in ion-implanted 3C-SiC probed by a monoenergetic positron beam. Jpn. J. Appl. Phys. Part 1 Regul. Pap. Short Notes Rev. Pap..

[33] Krause-Rehberg R., Leipner H.S. (1999). Positron Annihilation in Semiconductors-Defect Studies.

